# Progression of clinical diagnostic features and cognitive decline in mild cognitive impairment with Lewy bodies

**DOI:** 10.1017/S0033291725100895

**Published:** 2025-06-24

**Authors:** Calum Alexander Hamilton, Paul C. Donaghy, John-Paul Taylor, Joanna Ciafone, Rory Durcan, Michael Firbank, Gemma Greenfinch, Louise Allan, John O’Brien, Alan Thomas

**Affiliations:** 1 Translational and Clinical Research Institute, Newcastle University, Newcastle upon Tyne, UK; 2 Institute of Nuclear Medicine, University College London Hospitals, London, UK; 3 Centre for Research in Ageing and Cognitive Health, University of Exeter, Exeter, UK; 4 Department of Psychiatry, School of Clinical Medicine, University of Cambridge, Cambridge, UK

**Keywords:** dementia with Lewy bodies, disease progression, mild cognitive impairment, visual hallucinations

## Abstract

**Background:**

Mild cognitive impairment with Lewy bodies (MCI-LB) may be identified prospectively based on the presence of cognitive impairment and several core clinical features (visual hallucinations, cognitive fluctuations, parkinsonism, and REM sleep behavior disorder). MCI-LB may vary in its presenting features, which may reflect differences in underlying pathological pattern, severity, or comorbidity.

We aimed to assess how clinical features of MCI-LB accumulate over time, and whether this is associated with the rate of cognitive decline.

**Methods:**

In this cohort study, 74 individuals seen with MCI-LB prospectively underwent repeated annual cognitive and clinical assessment up to nine years. Relationships between clinical features (number of core features present and specific features present) and cognitive change on the Addenbrooke’s Cognitive Examination–Revised (ACE-R) were examined with time-varying mixed models. The accumulation of core clinical features over time was examined with a multi-state Markov model.

**Results:**

When an individual with MCI-LB endorsed more clinical features, they typically experienced a faster cognitive decline (ACE-R Score Difference β = −1.1 [−1.7 to −0.5]), specifically when experiencing visual hallucinations (β = −2.1 [−3.5 to −0.8]) or cognitive fluctuations (β = −3.4 [−4.8 to −2.1]).

Individuals with MCI-LB typically acquired more clinical features with the passage of time (25.5% [20.0–32.0%] one-year probability), limiting the prognostic utility of baseline-only features.

**Conclusions:**

The clinical presentation of MCI-LB may evolve over time. The accumulation of more clinical features of Lewy body disease, in particular visual hallucinations and cognitive fluctuations, may be associated with a worse prognosis in clinical settings.

## Introduction

Dementia with Lewy bodies (DLB) has four core clinical features: cognitive fluctuations, motor parkinsonism, complex visual hallucinations, and REM sleep behavior disorder (RBD) (McKeith et al., 2020). Core clinical features of Lewy body disease have a variable stage of onset, and not all are reported in all individuals (Donaghy et al., 2023). Indicative biomarkers of DLB are also used in diagnosis and highly specific to Lewy body disease, though they are sometimes normal in people with autopsy-confirmed Lewy body disease (Matsubara et al., 2022; Thomas et al., 2017). This variability in the onset of diagnostic features is particularly evident at the prodromal stage of mild cognitive impairment (MCI) with Lewy bodies (MCI-LB), when each individual clinical feature is less common than in DLB (Donaghy et al., 2018; Donaghy et al., 2022).

The four core clinical features are given equal weighting for diagnosis of DLB and MCI-LB, with the variability in onset giving rise to several possible phenotypes, e.g. some may be classified as MCI-LB with only a single core clinical feature and positivity on one or more indicative biomarker(s), other individuals may have several core clinical features but differ in which are present, and others may exhibit all four clinical features. These differences in individual symptomatology may reflect differences in underlying disease pattern, severity, or comorbidity (Ferreira et al., 2020; Inguanzo et al., 2023), which may have implications for prognosis, treatment and trials.

### Variable rates of progression in Lewy body disease

Whether individuals with such different clinical phenotypes in MCI-LB progress at different rates is important for providing accurate information about likely prognosis. Previous research examining the short-term progression of prospectively identified MCI-LB has found that presence of complex visual hallucinations is associated with progressive cognitive decline and greater risk of death (Hamilton et al., 2020; Hamilton et al., 2021), while cognitive fluctuations are associated with dementia onset (Hamilton et al., 2021). In contrast, though RBD is a strong risk factor for future DLB and Parkinson’s disease, individuals may present with RBD for several years before the onset of cognitive decline and dementia (Postuma et al., 2019).

This may suggest that differences in the rate of decline may be partially anticipated by differences in clinical presentation in MCI-LB. However, analysis of progression in retrospectively-identified MCI-LB (that is MCI cases eventually seen to develop DLB, but not necessarily recognized as MCI-LB prospectively) over the longer term has not found any associations between specific clinical features at first assessment, or the total burden of these, and rates of clinical progression (van de Beek et al., 2020).

Importantly, since MCI-LB is an evolving condition with clinical features developing over time (Donaghy et al., 2023), the combination of clinical diagnostic features at the point of initial diagnosis may be a less important predictor (since fewer features are present) than a later evolved presentation. We therefore aimed to assess whether differences in core clinical features and biomarkers were associated with longer-term disease progression in prospectively-identified MCI-LB, treating these as time-varying, and hypothesizing that:

1. The presence of a greater number of clinical diagnostic features would be associated with a faster rate of cognitive decline
2. The presence of visual hallucinations and/or cognitive fluctuations specifically would be associated with a faster rate of decline

## Method

Data were drawn from two prospective longitudinal studies of MCI-LB, with recruitment and assessment being reported several times previously (Donaghy et al., 2018; Donaghy et al., 2022), and briefly summarized below. Recruitment for these studies began in 2013 and ran until December 2019, with data locked for analysis in December 2023. There was therefore at least four years of follow-up available for all participants not withdrawn or lost to follow-up, and up to 10 years in some cases.

### Participants

People with a health service diagnosis of MCI were identified from older person’s medical services in Northeast England. They were required to be aged ≥60, free from dementia at baseline, medically stable, and without pre-existing Parkinson’s disease (that is any parkinsonism occurring >12 months prior to onset of cognitive symptoms) (Donaghy et al., 2018, 2022). Other core or supportive features of DLB were permitted to be present prior to diagnosis of MCI, as previously reported (Donaghy et al., 2023).

All participants provided written informed consent to participate.

### Assessment

Participants underwent neuropsychological assessment at baseline and annual follow-ups with the Addenbrooke’s Cognitive Examination–Revised (ACE-R) as the primary global cognitive outcome.

Participants and caregivers (where available) also underwent a detailed clinical interview at baseline and annual follow-up, providing information on the presence or absence of the four core clinical features of DLB. These were used to classify MCI as MCI-LB alongside indicative biomarkers (see below) and for analyses of different clinical phenotypes in this work.

Clinical features were assessed through a semi-structured interview guided by validated scales: the Clinician Assessment of Fluctuation and Dementia Cognitive Fluctuation Scale; the North East Visual Hallucination Inventory; the Unified Parkinson’s Disease Rating Scale Part III (motor sub-scale); and the Mayo Sleep Questionnaire.

Assessment of clinical features was not based on outcome scores alone, but considered the overall clinical picture (e.g. whether reported features could be attributed to another disease or process, other than Lewy body disease).

### Diagnosis

A three-person panel of expert old age psychiatrists (AJT, JPT, PCD) reviewed clinical research notes from baseline and follow-up to provide consensus diagnosis of clinical severity (MCI or dementia) and DLB clinical feature presence.

#### Clinical severity

Health service diagnoses of MCI were ratified within the study at baseline, with any cases of dementia, subjective-only cognitive impairment (SCI), or non-Alzheimer’s disease (AD)/LB etiologies excluded, including suspected vascular or frontotemporal etiologies as reported previously (Donaghy et al., 2018, 2022). MCI and dementia diagnoses were operationalized according to current consensus clinical criteria for any-cause MCI (Albert et al., 2011) or dementia (McKhann et al., 2011).

MCI diagnoses were reassessed after each follow-up visit, with consideration of any clinical progression of MCI to dementia. New dementia cases after baseline were retained for analysis.

#### Imaging

All participants were offered dopaminergic ^123^I–2β-carbomethoxy-3β-(4-iodophenyl)-N-(3-fluoropropyl) nortropane (FP-CIT) imaging at baseline (Roberts, Donaghy et al., 2021). A subset (*n* = 35) consented to repeat FP-CIT imaging at follow-up, which is included in these analyses (Durcan et al., 2023). FP-CIT imaging was visually assessed and classified as normal/abnormal by an experienced panel of imaging analysts.

^123^I-metaiodobenzylguianidine (MIBG) cardiac scintigraphy was offered to participants from the latter of two cohorts at baseline (*n* = 30 MCI-LB) (Roberts, Durcan et al., 2021). MIBG abnormalities were quantified by the ratio of MIBG uptake to the heart vs the mediastinum (HMR). HMR < 1.85 were considered abnormal, with this value derived from local healthy older adults (Kane et al., 2019).

Both imaging results were incorporated into differential classifications of MCI.

#### Etiological classification

At baseline, MCI due to suspected frontotemporal, vascular, or other non-AD/LB etiology was excluded (Donaghy et al., 2018, 2022). Those remaining were differentially classified according to consensus research and clinical criteria:

MCI with no core clinical features of DLB, no indicative biomarkers, and “evidence of decline consistent with AD with no evidence for another aetiology” (Donaghy et al., 2022) was classified as MCI-AD.

MCI with one core clinical feature of DLB and no indicative biomarkers, or no core clinical features of DLB but one or more indicative biomarkers, was classified as possible MCI-LB.

MCI with one core clinical feature of DLB and one or more indicative biomarkers, or those with two or more core clinical features regardless of biomarker results, was classified as probable MCI-LB.

**Figure 1.**
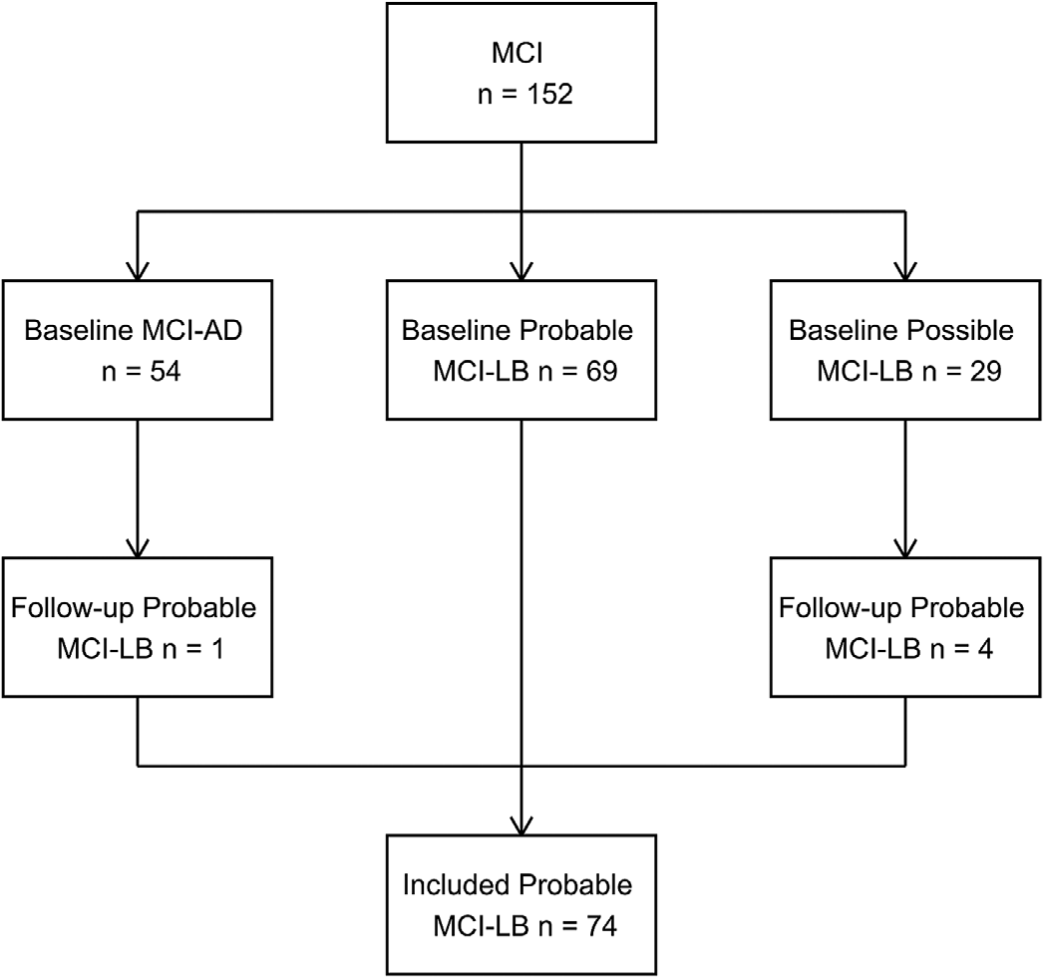
Prospective identification of included MCI-LB cases from baseline and follow-up MCI cohort.

As with MCI/dementia diagnoses, classifications were updated after follow-up as new clinical features were seen. All individuals with MCI who were identified either at baseline or follow-up to have probable MCI-LB were included in this analysis (see Figure 1).

### Analysis

The accumulation of clinical features of DLB in MCI-LB was examined using a multi-state Markov model allowing for sequential forwards (or backwards) progression of core clinical features of DLB. Forwards and backwards progressions were respectively constrained to be equal. This provides a highly flexible method of characterizing progression through ordinal disease stages with (e.g. Hamilton et al., 2021) or without assumption of monotonicity (as in this analysis), though other more complex patterns of disease progression may also be specified (Jackson, 2011).

Trajectories of cognitive performance were analyzed using linear mixed models adjusting for age, education, sex and socioeconomic disadvantage (English Indices of Multiple Deprivation), and participant-level correlations between baseline function and time slope. Clinical presentation (see below) was treated as a time-varying predictor with a time slope interaction and main effect at baseline.

In the primary analysis, core clinical features and indicative biomarkers were included as time varying predictors of cognitive performance. In three complementary models, we assessed the associations between cognitive decline and the overall (continuous) burden of clinical features, individual features/biomarkers, and hypothesized low (RBD/parkinsonism plus biomarker(s) only) vs high risk (any additional features) dichotomous phenotypes.

Sensitivity analyses examined the association of baseline-only, rather than time-varying, symptoms. For all models, non-linear time trends were tested for possible improvement in model fit based on the Bayesian Information Criterion.

### Ethics

The authors assert that all procedures contributing to this work comply with the ethical standards of the relevant national and institutional committees on human experimentation and with the Helsinki Declaration of 1975, as revised in 2008. All procedures involving human subjects/patients were approved by the National Research Ethics Service Committee North East – Newcastle and North Tyneside 2 (Approval nos. 12/NE/0290 and 15/NE/0420).

## Results

### Participant characteristics

*N* = 69 individuals were classified as probable MCI-LB initially, with an additional five at follow-up, providing *N* = 74 total cases of probable MCI-LB (see Figure 1). Baseline characteristics for these patients are provided in Table 1. Included participants had a median of three visits (upper and lower quartiles two to four), with a mean follow-up length of 2.6 years reflecting frequent conversion to dementia causing end of follow-up within three years (Hamilton, Donaghy et al., 2024), and a maximum follow-up of 8.8 years. At the point of initial assessment, MCI-LB cases reported having experienced their memory symptoms for a median of three years (Donaghy et al., 2023).

**Table 1.** Baseline characteristics of probable MCI-LB cases

Variable	Probable MCI-LB *N* = 74^a^
ACE-R total score	80.1 (9.7)
UPDRS-III score	24.3 (15.2)
Female	18/74 (24%)
Age	74.9 (7.2)
Years in education	11 [7–21]
Deprivation group (1–10)	5 [1–10]
Cognitive fluctuations	37/74 (50%)
Visual hallucinations	19/74 (26%)
Parkinsonism	29/74 (39%)
REM sleep behaviour disorder	45/74 (61%)
Abnormal FP-CIT	48/72 (67%)
Abnormal MIBG	17/30 (57%)

a Mean (SD); *n*/*N* (%); Median [Minimum–Maximum].

### Accumulation of clinical features

The clinical phenotype of MCI-LB typically progressed over time, with additional features emerging as MCI-LB developed towards DLB. Additional clinical features typically developed over 2–3 years, with an estimated one-year probability of 25.5% [20.0–32.0%] of endorsing any new feature(s) (see Supplementary Figure S1). Clinical features were occasionally, though infrequently, disendorsed at follow-up, with an estimated one-year probability of 5.7% [2.9–11.0%]. This occurred, for example, when a new informant provided better quality information than was available previously. Features which previously had good information for being present but appeared to have resolved (e.g. due to good response to medication) were treated as still being present.

Participant-level data on variation in core clinical features are presented in Supplementary Figure S2.

### Longitudinal change in cognitive function

**Figure 2.**
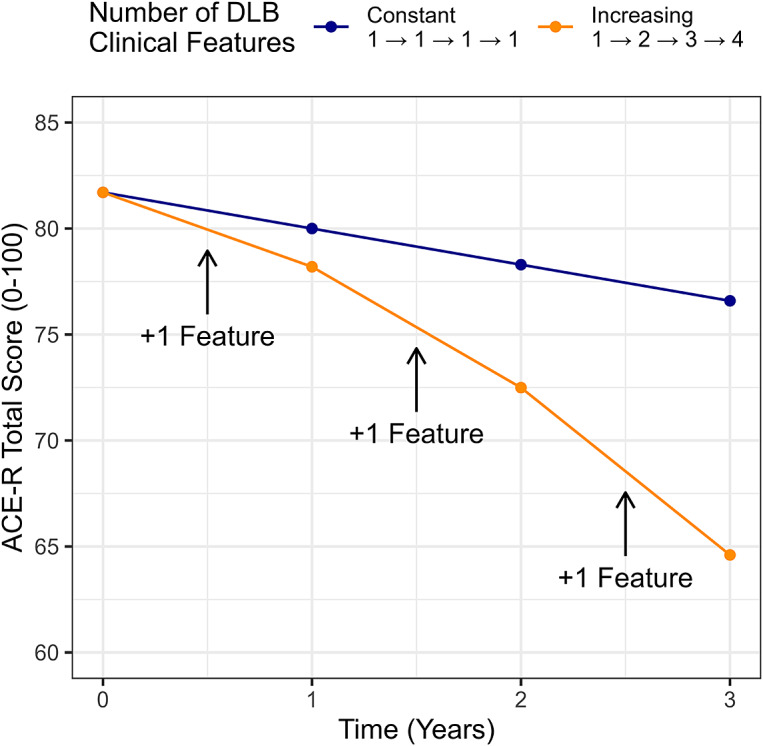
Model-derived predicted cognitive trajectories for an individual with a constant phenotype in MCI-LB vs MCI-LB progressively accumulating additional clinical features between observations.

Overall, cases of MCI-LB declined at an average rate of −2.9 [−3.9 to −1.9] points per year on the ACE-R (*p* < 0.001). This decline was significantly steeper in those with more clinical features of DLB (Estimate = −1.1 [−1.7 to −0.5] ACE-R points lost annually per feature, *p* < 0.001). The accumulation of features over time was therefore associated with steeper cognitive decline (see Figure 2 for covariate-adjusted conditional estimates for the median participant).

Complex visual hallucinations and cognitive fluctuations were specifically associated with more rapid cognitive decline within MCI-LB in individual models (see Table 2). In a multivariable model including these two predictors, cognitive fluctuations remained as the strongest predictor of greater cognitive decline. Consequently, as hypothesized, MCI-LB with an overall phenotype characterized by visual hallucinations and/or cognitive fluctuations therefore had significantly greater decline in cognitive function year-on-year than MCI-LB characterized by parkinsonism and/or RBD with or without abnormal biomarkers (Estimate = −2.8 per year [−4.1 to −1.5], *p* < 0.001).

**Table 2.** Associations between time-varying individual clinical features/biomarkers of LBD in MCI and annual change in ACE-R scores

Feature/biomarker	ACE-R change difference vs absent	*p* value
Cognitive fluctuations	−3.4 [−4.8 to −2.1]	<0.001
Visual hallucinations	−2.1 [−3.5 to −0.8]	0.002
Parkinsonism	−0.7 [−2.1 to 0.7]	0.31
REM sleep behaviour disorder	1.6 [−0.7 to 4.0]	0.182
Abnormal FPCIT imaging	−0.8 [−2.4 to 0.8]	0.32
Abnormal MIBG imaging	−0.1 [−1.9 to 1.7]	0.891

*Note:* Estimated difference [95% confidence interval].

**Figure 3.**
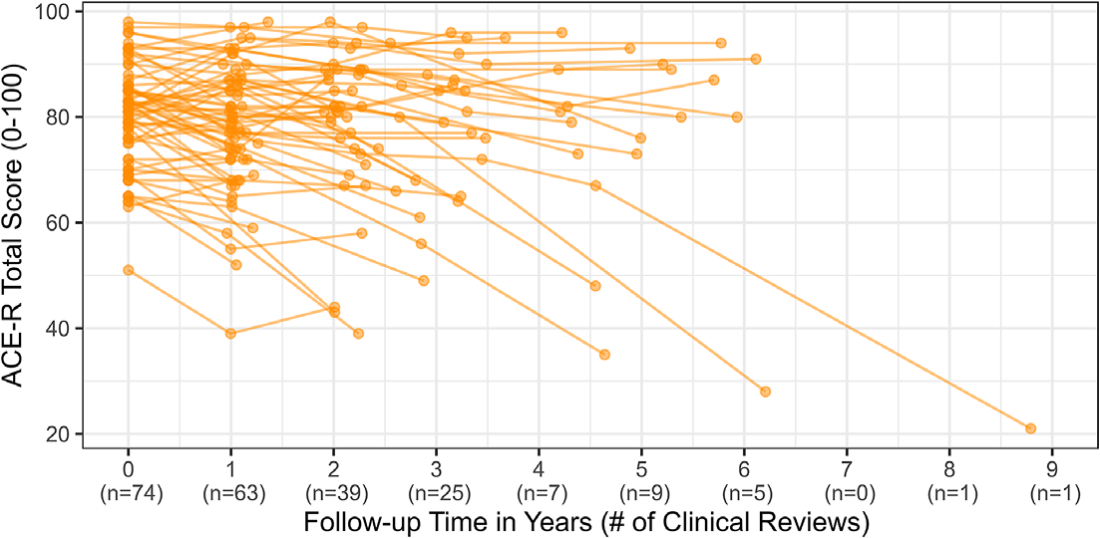
Raw ACE-R scores and clinical reviews across time.

Raw observations of cognitive outcomes are displayed in Figure 3.

### Sensitivity analyses

We examined whether the baseline-only phenotype was predictive of longer-term cognitive decline, restricting clinical features to be time invariant. In contrast to the time-varying phenotype, baseline clinical features alone were a poor predictor of differences in rate of long-term decline: overall count of features (Estimate = −0.4 [−1.5 to 0.7], *p* = .446) was not significantly associated with the rate of cognitive decline, nor was the overall baseline ‘high risk’ phenotype identified above (Estimate = −0.6 [−2.6 to 1.4], *p* = .572), which occurred in 42/74 individuals at baseline (57%).

Correspondingly, none of cognitive fluctuations (Estimate = −0.4 [−2.4 to 1.5], *p* = 0.687), visual hallucinations (Estimate = −2.0 [−4.4 to 0.3], *p* = 0.098), parkinsonism (Estimate = −0.2 [−2.3 to 2.0], *p* = 0.851), or RBD (Estimate = 0.6 [−1.5 to 2.7], *p* = 0.575) at baseline were associated with significantly better or worse longer-term rates of decline.

In all models, accounting for non-linear secular time trends did not improve model fit.

## Discussion

We examined whether the clinical phenotype observed in MCI-LB would be associated with disease progression over time, testing the hypotheses that:

1. MCI-LB cases with more core clinical features would progress faster over time
2. MCI-LB cases with cognitive fluctuations and/or visual hallucinations specifically would progress faster over time

We found that the clinical phenotype in MCI-LB was associated with rates of cognitive progression over time. Individuals with more core clinical features, or those with visual hallucinations or cognitive fluctuations, in particular, typically showed a greater decline in performance over time.

### Past research

Previous work including a subset of this cohort have indicated that, in the shorter term, visual hallucinations at baseline may be predictive of an overall declining cognitive trajectory (Hamilton et al., 2020), and as a time-varying covariate, are associated with risk of adverse outcome (death/dementia) (Hamilton et al., 2021). The presence of cognitive fluctuations and the increasing presence of any DLB clinical features overall were also associated with progression to dementia over the shorter period (Hamilton et al., 2021).

However, previous analyses have not identified a significant association between specific clinical features, or overall burden of these, at first assessment and rates of progression to dementia in retrospectively identified MCI-LB (van de Beek et al., 2020). Our study confirms such findings that baseline features alone do not appear to be predictive but builds on this, suggesting that the increasing presence of core features over the course of MCI-LB is associated with the rate of cognitive decline.

### Implications

The four core clinical features of DLB receive equal diagnostic weighting for DLB and prodromal DLB (IG et al., 2017; McKeith et al., 2020). However, our findings add to an emerging body of research suggesting that these may differ in their association with disease progression (Hamilton et al., 2020, 2021). Individuals with MCI-LB characterized by RBD or parkinsonism and biomarker abnormalities are highly likely to have underlying Lewy body disease (Boeve et al., 2013; Matsubara et al., 2022; Thomas et al., 2017), but do not consistently decline on average in cognitive testing. While many do endorse additional clinical features at follow-up, with a corresponding decline in cognitive performance, others may remain stable with this RBD-only phenotype for several years. This phenotype may be typical of those at an early stage of Lewy body disease, or with a less severe pattern of underlying pathology, but is consistent with the known long lead-in of idiopathic RBD prior to onset of DLB or PD (Claassen et al., 2010), and a relatively long period of normal cognition in PD (Lawson et al., 2021).

In contrast, MCI-LB characterized by the presence of cognitive fluctuations and/or visual hallucinations appears to decline more rapidly than those without these features. This may support that these symptoms typically occur later in the disease course closer to onset of dementia, or that these reflect more severe underlying patterns of Lewy body and/or Alzheimer’s pathological changes (Colloby et al., 2017; Ferreira et al., 2020; Inguanzo et al., 2023).

In contrast to the time-varying phenotype, baseline-only phenotype was not clearly predictive of long-term progression rate, which may reflect the relatively frequent emergence of new clinical features over the course of follow-up. The clinical phenotype of MCI-LB should not therefore be seen as fixed, but rather evolving over the course of MCI, requiring regular re-assessment of clinical features. Emergence of new features may be indicative of underlying disease progression, and associated with greater decline in cognitive function in parallel. This suggests that there may be a benefit in making sure people with MCI-LB and their caregivers are aware of these clinical features so that they are equipped to recognize and report any emergence of these.

### Strengths and limitations

This work benefits from a relatively long follow-up, approximately annually, of two prospective cohorts of MCI-LB enabling longitudinally emerging phenotype, replicating how this condition is seen to develop in real world settings.

A caveat to our finding that newly emergent clinical features predict decline is that such features do not emerge exactly at the point of cognitive re-assessment and may have first become evident at any point in time between two consecutive cognitive testing times. This likely varies between individuals and leaves open the possibility that expedited cognitive decline may precede, rather than follow, emergence of new risk-associated clinical features.

Relatedly, we did not find baseline features alone to predict future cognitive decline, suggesting limited clinical utility of baseline features alone as an indicator of longer-term prognosis. This could reflect several causes: the clinical phenotype may still be emerging in newly-diagnosed MCI, as reflected by relatively high accumulation of clinical features annually in this group, with subsequent re-assessments providing a clearer indication of the prognosis. Rather than a predictor of cognitive decline, the clinical phenotype may be a co-marker of disease progression, with clinical features emerging in parallel to, rather than prior to, progressive cognitive decline. Alternatively, this could reflect a lack of statistical power given the relative infrequency of some clinical features, in particular visual hallucinations, at baseline in MCI-LB (Donaghy et al., 2018, 2022). The annual re-assessment of participants limited our ability to assess whether newly emergent clinical features co-occurred with cognitive decline, or anticipated these. Future studies incorporating more intensive longitudinal cognitive assessment may be able to examine this in more depth.

Our finding that baseline features did not predict longer-term cognitive decline also points to an important limitation in interpreting our imaging biomarker findings, namely that these were largely restricted to baseline only. Whilst we were able to repeatedly assess clinical features we could not do so annually for these biomarkers and it is therefore possible that these too might have been predictors if such repeat assessment had been possible. However, in a sub-sample of these cohorts with repeat FP-CIT imaging, the annual rate of change in this biomarker was statistically significant but clinically of limited utility (Durcan et al., 2023), suggesting that repeated acquisition of such biomarkers may not provide any additional predictive power for cognitive decline.

Entry into, and exit from, this study were based on the time of diagnosis of MCI and dementia, respectively. However, both of these landmarks represent arbitrary points in a continuous process of progressive decline. Some individuals will have entered the study at a later point in their disease process, while others will have entered comparatively earlier. While some methods (e.g. the multi-state Markov model) inherently accounted for differences in baseline staging, others (e.g. the longitudinal analysis of cognitive decline) do not. Core features and cognitive impairments may have developed at different rates prior to recognition of MCI, and after development of dementia. We sought additional information after onset of dementia for a subset of cases (*n* = 6) but did not have information available on subsequent decline for the majority of this cohort, limiting our ability to assess longer-term progression.

The ACE-R was the primary cognitive outcome measure in this study. While this did show significant decline overall, this may lack sensitivity to decline in MCI-LB and DLB, with high episodic memory weighting and comparatively little weighting on visuospatial and executive functions (Tsamakis & Mueller, 2021). Individual differences in rates of decline could therefore be explained not by differences in global progression, but rather by differing patterns of neurocognitive impairment which align with the domains to which the ACE-R is most sensitive.

Both cognitive outcomes and time-varying clinical presentation are linked by their shared relationship with the passage of time in this study. This could be interpreted to represent an apparent issue of circularity, whereby greater follow-up naturally gives rise to recognition of new clinical features and deterioration in cognitive function. However, we do note prognostic differences between clinical features, not just the accumulation of these, which supports an association independent from the passage of time alone.

Cognitive fluctuations were found to hold the strongest association with progressive decline. However, these are unlike other features in that they directly reflect cognitive function, though judgement of their presence is not made with reference to cognitive scores. It therefore remains possible that fluctuations directly impact on cognitive performance during testing, rather than reflecting underlying disease stage, pattern, or progression. While we allowed for a cross-sectional main effect which could account for this effect, but was not supported by the data to hand, previous analyses of more detailed neurocognitive tests from a subset of this cohort have suggested that cognitive fluctuations may be associated with greater errors in sustaining attention in MCI-LB (Hamilton, Gallagher et al., 2024). This therefore warrants further exploration.

## Conclusions

MCI-LB cases typically accumulate additional clinical features with the passage of time. This accumulation of features may be a marker of disease progression, being associated with faster cognitive decline over time. Visual hallucinations and cognitive fluctuations appear to be particularly associated with disease progression in contrast to parkinsonism and/or RBD.

## Supporting information

Hamilton et al. supplementary material Hamilton et al. supplementary material

## Data Availability

Data from the cohorts used in these analyses are available through the Dementias Platform UK data portal.

## Supplementary material

The supplementary material for this article can be found at http://doi.org/10.1017/S0033291725100895.
