# Supplementary figures and images for: Progression of clinical diagnostic features and cognitive decline in mild cognitive impairment with Lewy bodies
Source: Psychol Med. 2025 Jun 24;55:e175. doi: 10.1017/S0033291725100895 (PMC13112294; doi:10.1017/S0033291725100895)

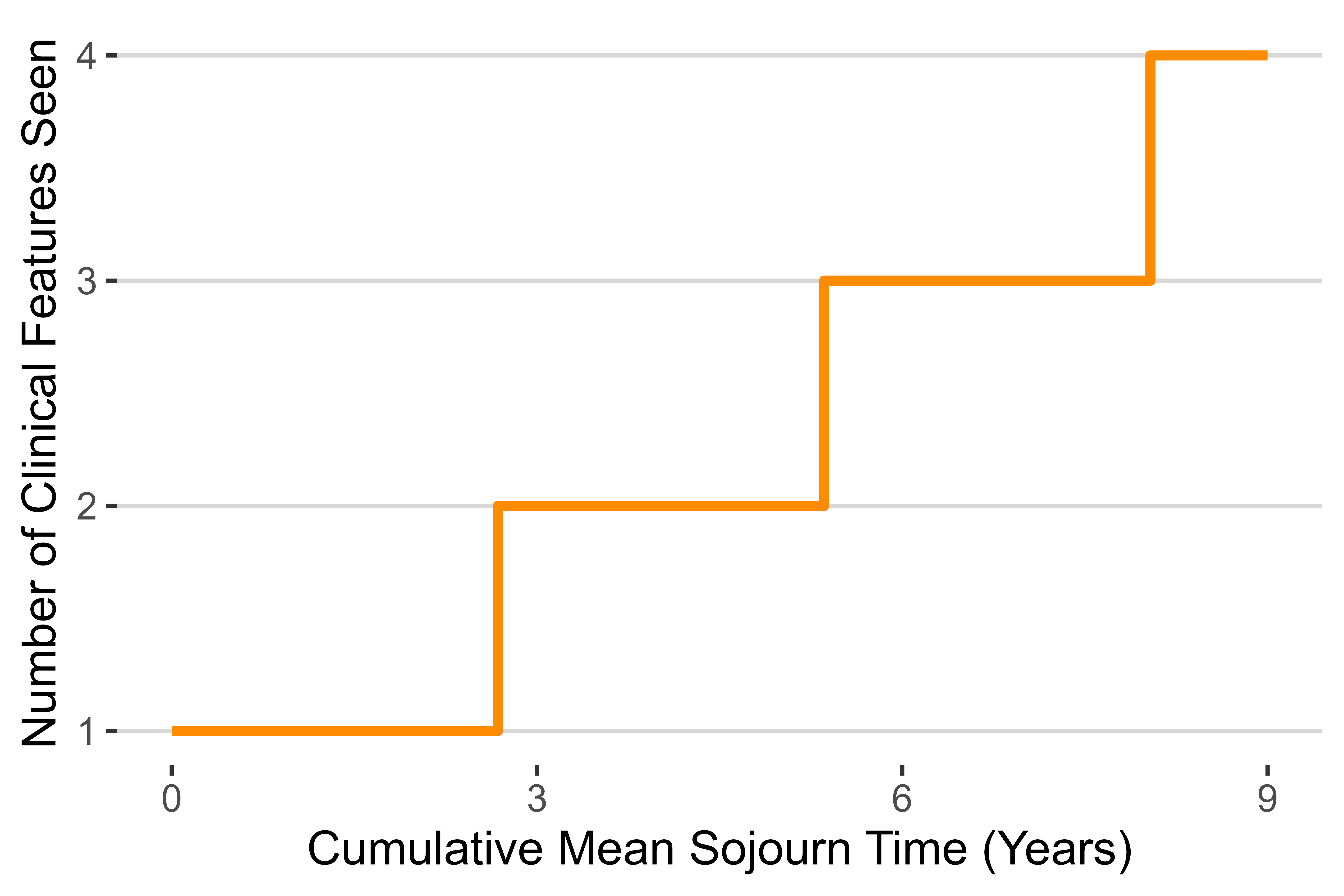

Supplement: Hamilton et al. supplementary material [file S0033291725100895sup001.zip › SuppFigS1.tiff]
